# Protocol for a low-volume, direct analysis urine preparation procedure for non-targeted GC-MS metabolomics

**DOI:** 10.1016/j.xpro.2024.103449

**Published:** 2024-11-15

**Authors:** Bianca Allen, Laneke Luies

**Affiliations:** 1 Focus Area Human Metabolomics, North-West University (Potchefstroom Campus), Potchefstroom, North West 2520, South Africa

**Keywords:** Chemistry, Health Sciences, Mass Spectrometry, Metabolism, Metabolomics

## Abstract

We present a low-volume, direct analysis protocol for non-targeted gas chromatography-mass spectrometry (GC-MS) metabolomics, using 100 μL of urine. The steps include sample collection, stock solution preparation, metabolite extraction, two-step derivation with a drying phase, and analysis via two-dimensional GC time-of-flight MS (GCxGC-TOFMS). This protocol improves the efficiency and thoroughness of urinary metabolite analysis, contributing to advancements in metabolomics research, disease diagnosis, and biomarker discovery.

For complete details on the use and execution of this protocol, please refer to Olivier et al.^1^

## Highlights

• Collection of urine samples with ethical handling and proper storage
• Preparation of laboratory and instruments for optimal GC-MS performance
• Steps for conducting two-step derivatization are methoximation followed by silylation
• Protocol for comprehensive GCxGC-TOFMS analysis for metabolite detection

## Abstract (editor highlights)

Publisher’s note: Undertaking any experimental protocol requires adherence to local institutional guidelines for laboratory safety and ethics.

## Graphical abstract

**Figure:**
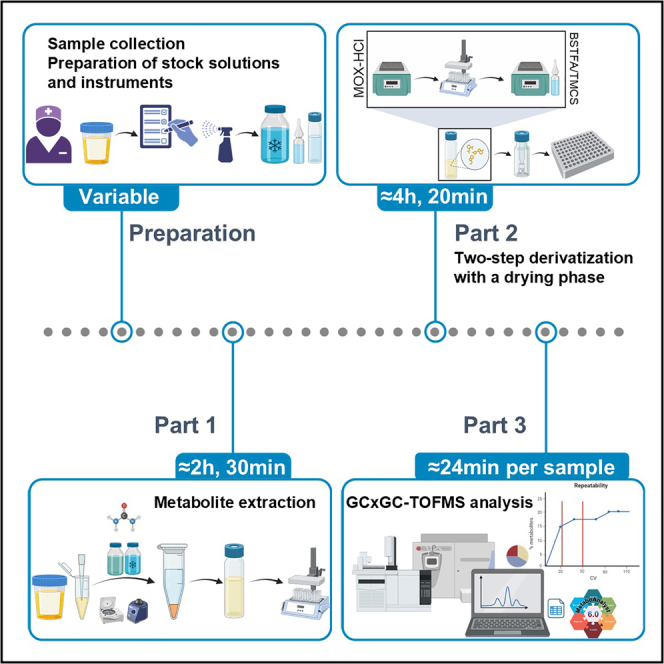

## Before you begin

Metabolomics investigates dynamic metabolic changes in response to genetic modifications or physiological stimuli, analyzing the metabolome, the complete set of small molecules present in a sample.^2^ Urine, reflecting normal and pathological processes, is valuable for metabolomics due to ease of collection and handling.^3^ Gas chromatography mass spectrometry (GC-MS) is preferred for its affordability, sensitivity, and compound identification capabilities. To this end, non-targeted GC-MS metabolomics aims to investigate multiple compound classes at various concentrations, hence optimal compound extraction is essential.^4^ An extraction protocol that can simultaneously identify all the metabolites in a urine sample is not yet available, mostly due to the complexities and the variety of physiochemical properties of metabolites. However, the following direct analysis (DA) protocol was previously compared to various other protocols, and showed superior repeatability, metabolome coverage, and metabolite recovery.^1^ This protocol was also subsequently used to extract urine samples for disease characterization studies.^5^ It has the advantage of requiring little sample volume (only 100 μL), have few analytical steps (which are more time efficient, cost-effective, and repeatable), and can extract as much of the metabolome as possible (over 500 compounds), making it ideal for non-targeted urine GC-MS analysis.

### Institutional permissions

Notably, all experiments on live vertebrates or higher invertebrates must be performed in accordance with relevant institutional and national guidelines and regulations. All previous investigations relating to the current protocol were done according to the Declaration of Helsinki and International Conference of Harmonization guidelines. Ethical approval was obtained from the Human Research Ethics Committee of the North-West University (ethics no. NWU-00355-20-A1 and NWU-00355-20-A1–04). Herewith, readers are reminded to acquire permissions from their relevant institutions.

### Preparation 1: Obtain urine samples

**Timing: N/A**

This section outlines essential procedures for obtaining, handling, and storing urine samples to ensure their suitability for metabolomics analysis. By adhering to these guidelines, researchers can maintain the integrity and reliability of the urine samples, allowing for accurate downstream metabolomics investigations.

1. Human urine samples can be obtained from pathology laboratories, clinical, or hospitals, provided they are collected ethically, and patients provide written and informed consent. Similarly, animal urine samples can be sourced from veterinary clinics, research facilities, animal shelters, or farms. In all cases, samples must be collected in accordance with ethical guidelines, and the appropriate approvals and consents must be obtained from relevant authorities.

**CRITICAL:** Determine the creatinine values of each urine sample. Although this protocol does not require urine volume adjustment based on creatinine, the values will be needed for normalization after sample analysis.

***Note:*** There are no specific time-of-day requirements for urine collection, as this protocol accommodates variability in metabolite concentrations.

***Note:*** Urine samples should ideally be stored at −70°C or −80°C (long-term storage) to preserve metabolite integrity. However, it can be stored at −20°C for short durations without significant stability issues.^6^ It is recommended to aliquot samples before freezing to minimize the impact of freeze-thaw cycles, which can alter metabolite profiles. Fresh urine can be stored at 4°C for short periods (up to 12 h), but should be analyzed promptly to avoid degradation.

***Note:*** The protocol is suitable for both normal and diseased urine profiles. High protein content due to certain conditions is not an issue as a protein removal step is included. The protocol has also been successfully applied in infectious diseases studies.^5^ However, significantly diluted urine (e.g., from diabetes insipidus) may impact the protocol’s efficacy. In such cases, adjusting urine volume might be necessary to account for the dilution. Notably, the effect of highly diluted urine on protocol performance has not been investigated.

### Preparation 2: Preparing the laboratory and analytical instrument

**Timing: 2 days**

This section covers the preparation of the laboratory environment and analytical instruments for GC-MS analysis, ensuring optimal performance and minimizing contamination.

2. Ensure all equipment and consumables are available and reserved.
3. Conduct routine clean-up with a needle wash solution (see point 5) and perform a maintenance check on the GC-MS.

***Note:*** Conduct leak checks, tune checks, mass calibration, and run blank samples using an empty GC vial. Replace the liner and septum to prevent undesired reactions or adsorption. If using a two-dimensional GC system, defrost it before use. While these steps are largely described in system manuals provided with purchase, further general guidance can be accessed at https://go.leco.com/lecolearn.

4. Wipe benchtops, basins, and fume hood daily with 70% ethanol before and after use.
  a. The 70% ethanol can be prepared using the formula $C_{1}\;x\;V_{1}=C_{2}\;x\;V_{2}$, where C_1_ is the concentration of the starting ethanol (e.g., either 100% or 95%), C_2_ is the desired concentration (70%), and V_2_ is the final volume (1 L).
    i. For 100% ethanol, measure 700 mL of ethanol into a glass beaker and add distilled water to reach 1 L. Mix thoroughly.
    ii. For 95% ethanol, for example, using the same formula, measure 736.8 mL of 95% ethanol and add distilled water to 1 L. Mix thoroughly.
5. Prepare a needle wash solution by combining isopropanol and acetone in a 1:1 ratio in a 250 mL Schott bottle.

***Note:*** The solution can be stored indefinitely, typically in the fume hood for easy access.

6. Set the Techne Dri-block Heater with sample concentrator to 37°C. This heater, combined with nitrogen gas, facilitates solvent evaporation and sample concentration.

***Note:*** If multiple heating blocks are available, set one to 50°C for methoximation and another to 60°C for silylation. If using only one block, temperatures will need to be adjusted throughout the protocol.

***Note:*** Ensure that the correct size sample holding blocks are placed in the instrument.

**CRITICAL:** Uncapped samples should always be heated in the fume hood (not on the benchtop).

7. Place acetonitrile (ACN), with the lid sealed with parafilm, in a −20°C freezer for at least 20–30 min before the experiment. Ensure slightly more ACN is aliquoted than needed, keeping in mind that each sample will require 300 μL ACN. Thus, for 10 samples, place (10 + 1) × 300 μL = 3,300 μL = 3.3 mL into the freezer.

***Note:*** Remove the ACN from the freezer approximately 5 min before use (see Part 1, point 4) to avoid the lid freezing shut.

***Note:*** While Teflon-lined caps are a good alternative to prevent leakage, they can create a very tight seal, which may lead to pressure buildup if vapor is not adequately vented. This could result in the cap blowing off or causing leakage if the pressure exceeds the cap’s tolerance.

**CRITICAL:** Store the ACN container tightly closed in a dry, well-ventilated place, away from heat and ignition sources. Use it within 2–3 years of purchase, and inspect regularly for signs of deterioration or leakage.

8. Wash all glassware using phosphate-free soap such as Liquinox while wearing gloves:
  a. Rinse with clean tap water.
  b. Rinse five times with milli-Q water.
  c. Dry completely in a 90°C oven for 1 h.
9. Autoclave pipette tips (if non-filtered) to ensure sterility:
  a. Load the correct size tips into a pipette box and secure with autoclave tape by pressing down firmly.
  b. Set autoclave to 121°C and 1 bar pressure for a 20 min cycle. Use the dry cycle to prevent vapor issues.
  c. Allow the tips to cool inside the autoclave before using. Drying can also be done using an oven set at 60°C or less, for 30–60 min. Allow tips to cool completely to avoid condensation.
10. Clean the drying nozzles of the nitrogen dryer/sample concentrator to prevent contamination and prolong nozzle life:
  a. Remove individual nozzles from the nozzle head assembly and submerge them fully in a separately prepared needle wash solution (see point 5).
    ***Note:*** Allow the nozzles to soak in the solution for 8–12 h to remove contaminants.
    ***Note:*** Do not use the main needle wash solution for this step. Prepare an additional needle wash solution, or aliquot an appropriate volume of the cover the nozzles.
  b. After soaking, remove the nozzles from the solution and rinse thoroughly with milli-Q water.
  c. Wipe the exterior of the nozzles with a paper towel to remove any excess moisture.
  d. Ensure the nozzles are completely dry before placing them back into the nozzle head assembly.

### Preparation 3: Preparing stock solutions

**Timing: ∼1 h, 15 min**

This section provides detailed instructions for preparing essential solutions, focusing on proper handling and storage to maintain accuracy and reliability.

11. Prepare an internal standard solution:
  a. Prepare a 500 ppm stock solution:
    i. Weigh 25 mg of 3-phenylbutyric acid using a glass weighing boat, and transfer it to a 50 mL volumetric flask through its glass funnel.
    ii. Rinse the glass weighing boat twice with ∼2 mL milli-Q water.
    iii. Add milli-Q water to just below the 50 mL mark and swirl to dissolve.
    iv. Top up to the 50 mL mark with milli-Q water using a plastic dropper, cap, and shake gently.
      ***Note:*** If the solution does not dissolve, sonicate using an ultrasonic water bath sonicator for 20 min or until fully dissolved. Keep the volumetric flask capped during sonication to avoid contamination. If the solution still does not dissolve, refer to troubleshooting (problem 1) for guidance.
  b. Prepare a 50 ppm working solution:
    i. Pipette 5 mL of the 500 ppm solution into a 50 mL volumetric flask using a glass graduated pipette.
    ii. Fill to 50 mL with milli-Q water, using a plastic dropper for precise filling.
    iii. Transfer the solution to a clean, labeled 50 mL Schott bottle.
  c. Mark the bottles properly (contents, concentration, date, initials).
    ***Note:*** The 500 ppm stock solution and 50 ppm working solution can be stored at −80°C for up to 6 months or at −20°C for up to 1 month. If the solutions are older than this, prepare fresh to ensure optimal accuracy.
12. Prepare the methoxyamine hydrochloride (MOX-HCl; 20 mg/mL):
  a. Weigh 20 mg of MOX-HCl using a glass weighing boat.
  b. Dissolve in 1 mL of pyridine using a Hamilton syringe (in a fume hood). If it does not dissolve, refer to troubleshooting (problem 2) for guidance.
    ***Note:*** Although this protocol describes the use of a Hamilton syringe, any similar high-precision syringe for handling small volumes will be appropriate.
  c. Transfer to a clean, marked GC vial (if the final volume is less than 1.5 mL) and seal with parafilm.
    **CRITICAL:** MOX-HCl solution should be prepared fresh daily for optimal results, though it be stored up to a week in a desiccator at room temperature (20°C–22°C). Discard any leftover solution.
    **CRITICAL:** Clean and maintain the Hamilton syringe before and after each use by aspirating and discarding the needle wash three times. This ensures optimal performance and longevity by preventing sample residue from sticking.
    ***Note:*** Avoid wastage by preparing only the necessary volume of MOX-HCl. For example, 10 samples require 50 μL MOX-HCl each (see Part 2, point 9 below), thus 10 × 50 = 500 μL MOX-HCl. However, it is advisable to prepare slightly more than the exact volume needed (an additional 100 μL is recommended). Prepare 600 μL by dissolving 12 mg MOX-HCl in 600 μL pyridine. Avoid preparing less than 500 μL as handling very small volumes can be challenging.
    ***Note:*** This is a light-sensitive reaction. Switch off the fume hood light but keep the extractor fan on as per safety guidelines. Keep the solution away from light until ready to use. Use amber GC vials to further protect from light, though this is not strictly necessary due to the rapid nature of the protocol.
    ***Note:*** Discard expired MOX-HCl powder as it should be used within one year of purchase.
13. Prepare N,O-bis(trimethylsilyl)trifluoroacetamide (BSTFA) with 1% trimethylsilyl chloride (TMCS): This reagent is purchased “ready to use” in ampoules.
  a. Break the ampoule carefully and transfer the reagent into a GC vial using a clean Hamilton syringe.
  b. Cap the vial tightly and seal with parafilm to prevent evaporation.

***Note:*** When retrieving the solution, pierce the cap’s septum with a clean syringe instead of removing or puncturing the parafilm, to maintain sterility and prevent exposure to atmospheric moisture.

**CRITICAL:** Derivatization reagents (such as BSTFA/TMCS) are highly hygroscopic. Store in a refrigerator or a cool, dry place in a sealed container. Before use, let it equilibrate to room temperature (20°C–22°C) to prevent condensation. Never expose to air for prolonged periods, as it will compromise its effectiveness in derivatization.

## Key resources table

**Table:** 

REAGENT or RESOURCE	SOURCE	IDENTIFIER
**Biological samples**		

Urine samples	Human urine samples: pathology laboratories, clinical, hospitals, etc. Animal urine samples: veterinary clinics, research facilities, animal shelters, or farms.	N/A

**Chemicals, peptides, and recombinant proteins**		

3-Phenylbutyric acid, 98%	Merck/Sigma-Aldrich	CAS# 4593-90-2; Product Code: 116807
Acetone, ACS reagent, ≥99.5%	Merck/Sigma-Aldrich	CAS# 67-64-1; Product Code: 179124
Acetonitrile, anhydrous, 99.8%	Merck/Sigma-Aldrich	CAS# 75-05-8; Product Code: 271004
Ethanol, 99.9% 2.5 L	Merck/Sigma-Aldrich	CAS# 64-17-5; Product Code: 1009862500
Fatty acyl methyl esters (FAMEs)	Merck/Sigma-Aldrich	EC# 200-838-9; Product Code: CRM47885
Isopropyl alcohol, ACS Reagent, for HPLC	Merck/Sigma-Aldrich	CAS# 67-63-0; Product Code: 190764
Liquinox	Merck/Sigma-Aldrich	Product Code: Z742916
Methoxyamine hydrochloride (MOX-HCl) 98%	Merck/Sigma-Aldrich	CAS# 593-56-6; Product Code: 226904
N,O-bis(trimethylsilyl)trifluoroacetamide (BSTFA) with 1% trimethylsilyl chloride (TMCS)	Merck/Sigma-Aldrich	CAS# 25561-30-2; Product Code: 15238-10X1ML
Pyridine, anhydrous, 99.8%	Merck/Sigma-Aldrich	CAS# 110-86-1; Product Code: 270970
Urease	Merck/Sigma-Aldrich	CAS# 9002-13-5; Product Code: U4002

**Deposited data**		

Raw and analyzed data	This paper and Olivier et al.^1^	BioStudies database: S-BSST1138

**Software and algorithms**		

MetaboAnalyst	Online resource	https://www.metaboanalyst.ca/

**Other**		

Eppendorf Protein LoBind tubes, 2.0 mL	Merck/Sigma-Aldrich	Product Code: EP0030108132-100EA
GC vial caps, short screw, 9 mm Blue PTFE/Sil, pack of 100	LECO	Product Code: YY-RES-24485
GC vial inserts, 50 μL glass w/polypropylene bottom spring, pack of 1,000	LECO	Product Code: YY-RES-21782
GC vial, short cap, 2 mL, clear 12 × 32, 9 mm, screw thread finish, pack of 100	LECO	Product Code: YY-RES-21154
Glass Pasteur pipettes, short capillary tip, approx. 2 mL withdraw volume, soda-lime glass	Merck/Sigma-Aldrich	Product Code: Z627992-1000EA
Rxi-17Sil MS capillary column	LECO	Product Code: YY-RES-15123
Rxi-5Sil MS capillary column	LECO	Product Code: YY-RES-13623
Hamilton syringe, or alternative syringe: 100 μL removable needle (RN) gastight 22ga/51 mm/LC	Stargate Scientific	Product Code: 005312
Parafilm M laboratory sealing film	Merck/Sigma-Aldrich	Product Code: HS234526B
Weighing scoop, 3 mL glass	Merck/Sigma-Aldrich	Product Code: SLW2115/02M
Volumetric flask, 50 mL glass	Merck/Sigma-Aldrich	Product Code: Z326895
Funnel, short stem glass	Merck/Sigma-Aldrich	Product Code: BR145535
Schott bottle, 100 mL	Merck/Sigma-Aldrich	Product Code: BR122538
Schott bottle, 250 mL	Merck/Sigma-Aldrich	Product Code: BR122548
Pegasus 4D-C GCxGC-TOFMS	LECO	Model: Pegasus IV Mass Spectrometer, housing an Agilent 7890A modified with a LECO Thermal Modulator II cryostatic cooling device
Multipurpose sampler	Gerstel	Model: MPS2 rail autosampler
Centrifuge	Thermo Scientific	Description/code: 75009770
Vortex (mini mixer)	Lasec	Description/code: IMIUMIX-25P
Techne Dri-block heater with sample concentrator	Merck/Sigma-Aldrich	Description/code: Z381330
Analytical balance	Shimadzu	Description/code: AP225W
Autoclave	ALP Lasec	Description/code: IALPKTR-3065A
Ultrasonic cleaner (200 W, 6.5 L)	RS Pro	Description/code: 703
Milli-Q Purification System	Merck Millipore	Model: Milli-Q® IQ 7000 Ultrapure Lab Water System

## Step-by-step method details

In metabolomics analyses, including quality control (QC) samples, system blanks (SBs), and extraction blanks (EBs) are essential for maintaining data quality and integrity. QC samples, created by pooling small volumes from all urine samples into one vial, serve as a reference standard throughout the analysis. They are used to condition the analytical platform, measure reproducibility within the study, and correct for systematic errors. SBs are performed by running a blank gradient, without any sample or reagents, to detect impurities within the separation system. EBs simulate the sample preparation process without including any biological material, revealing contaminants or artifacts introduced during handling and processing. These blank samples are vital for distinguishing and eliminating non-biological signals, ensuring precise data interpretation.^7^

The time required for the protocol depends largely on the number of samples being processed. The provided times are based on processing approximately 20 samples, which constitutes a typical batch size that can be analyzed on a GC within a 24 h period using this protocol. Each batch, defined as the number of samples handled and analyzed within a single day, should be analyzed within 24 h after derivatization, as specified in Part 2. Processing numerous samples in batches over several days helps to efficiently manage the analytical workload. By including quality assurance samples in each batch, researchers can identify and address any batch effects, thereby maintaining high data quality and minimizing analytical variability. We also recommend that each batch is systematically arranged with SBs, EBs, QCs, and randomized experimental samples to ensure that each batch represents the entire cohort. Furthermore, fatty acyl methyl esters (FAMEs) can be injected before and after each analytical batch as retention index standards.

### Part 1: Sample preparation

**Timing: ∼2 h, 30 min**

**Figure 1:**
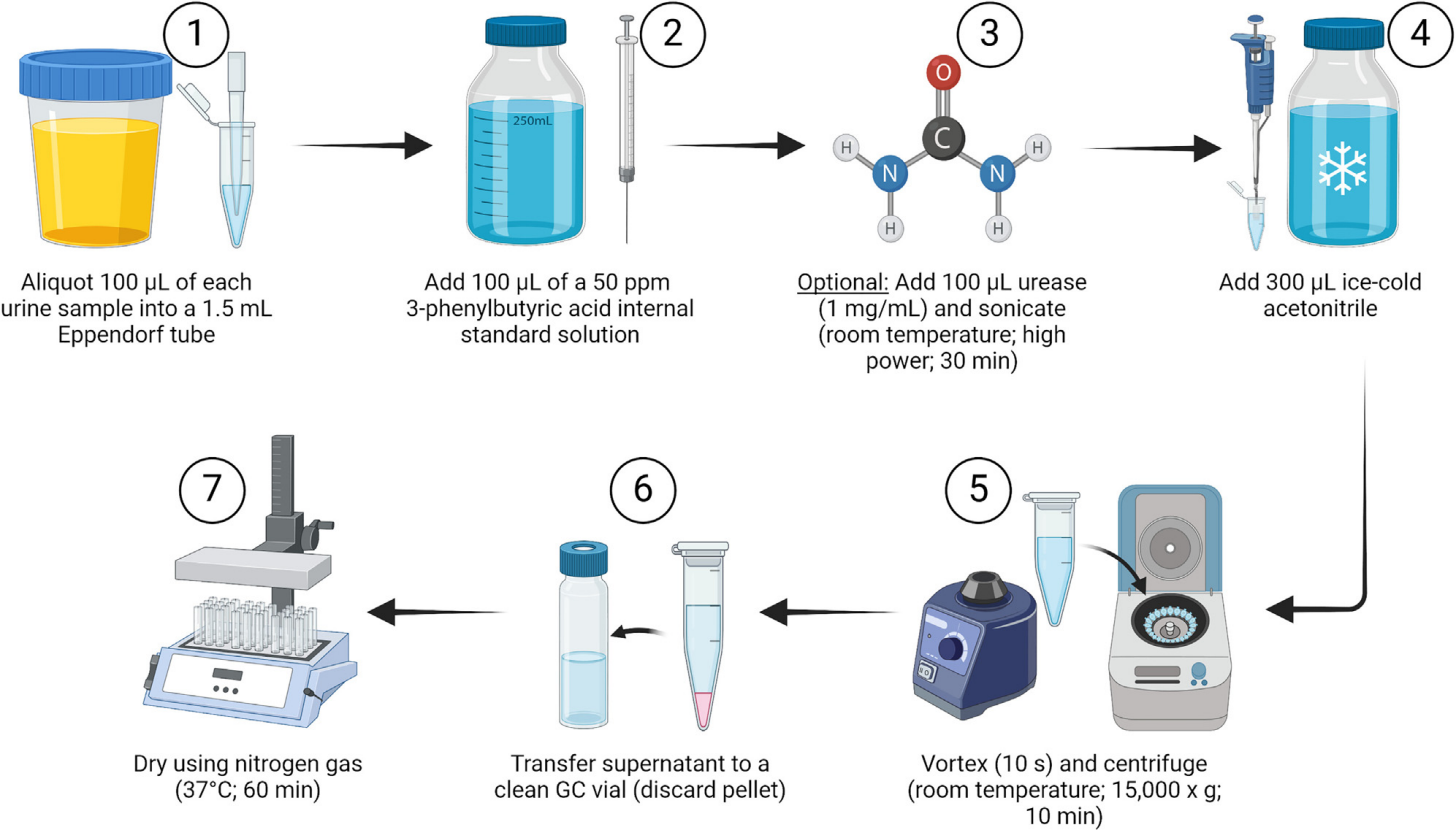
Overview of the urine sample preparation protocol This protocol includes the following steps: (1) aliquot 100 μL of urine into Eppendorf tubes, (2) add 100 μL of a 50 ppm internal standard solution, (3) optionally add 100 μL of urease solution and sonicate for 30 min, (4) add 300 μL of ice-cold acetonitrile, (5) vortex briefly and centrifuge at 15,000 × *g* for 10 min, (6) collect the supernatant, and (7) dry the samples completely under a light stream of nitrogen gas at 37°C. [Created using BioRender.com].

Figure 1 summarizes the sample preparation workflow, comprising the following steps, which are performed at room temperature (20°C–22°C).

1. Aliquot 100 μL of each urine sample into a 1.5 mL LoBind Eppendorf microcentrifuge tube under sterile conditions. Ensure the sample is properly mixed before transferring an aliquot by vortexing or pipetting up and down.
2. Label each sample clearly.

***Note:*** Urine samples can be stored at −20°C for short durations without significant stability issues. For extended storage, it is recommended to store at −70°C or −80°C to better preserve sample integrity.^6^ When thawing sample aliquots, do so at room temperature (20°C–22°C). Once thawed, vortex briefly (10 s) with pulse mode to resuspend any settled or precipitated materials in the urine sample, creating a uniform suspension (without causing excessive foaming or splashing). Then centrifuge the samples at 15,000 × *g* for 5 min at room temperature (20°C–22°C) to eliminate any crystals.

3. Add 100 μL of the 50 ppm 3-phenylbutyric acid internal standard solution, using a clean Hamilton syringe.
  ***Note:*** A Hamilton syringe, or a similar high-precision syringe, is used to add the internal standard solution due to its superior accuracy and precision for handling small volumes. This ensures consistent and reliable measurements. While micropipettes can be used as an alternative, high-precision syringes are preferred for enhanced accuracy in critical applications.
  ***Optional:*** One challenge in non-targeted metabolomics using urine samples is the presence of urea, which can mask other metabolites during analysis or interfere with derivatization. Although urease effectively reduces the urea content, it generally has a negative impact on overall metabolic signal quality. Therefore, use urease pre-treatment only when necessary for specific urea-related analysis. If urease pre-treatment is needed, sonication is preferable to heating.^1^
  a. Add 100 μL urease (1 mg/mL) and sonicate the samples for 30 min at room temperature (20°C–22°C) with high (005) power to ensure an adequate reaction.
    ***Note:*** To prepare urease, dissolve 1 mg of urease in 1 mL of milli-Q water. The volume needed will depend on the number of samples being processed; adjust the quantity accordingly.
    ***Note:*** If the solution does not fully dissolve, sonicate at room temperature (20°C–22°C) for up to 30 min. To prevent denaturation, do not use heating during sonication, as urease begins to denature at temperatures above 45°C.
    ***Note:*** Store the prepared urease solution at 4°C, where it remains stable for up to 28 days without significant loss of activity. Avoid storing urease solutions at temperatures below 0°C.
4. Add 300 μL ice-cold ACN using a pipette. This value is typically three times the sample volume.
5. Vortex briefly (10 s) the samples to ensure sufficient mixing of ACN, but without causing excessive foaming or splashing.
6. Centrifuge samples at room temperature (no additional settings) at 15,000 × *g* for 10 min.

***Note:*** Place all the tubes with the hinges facing outward so that the pellet forms on the same side of all tubes, facilitating the removal of the supernatant in the next step.

7. Collect the supernatant (i.e., the top layer) using a glass Pasteur pipette and transfer it to a clearly labeled GC vial. Discard the pellet.

***Note:*** Glass Pasteur pipettes require the use of a rubber bulb. It is best to immerse the pipette tip just below the liquid’s surface rather than immersing the pipette as deep as possible. This helps avoid disturbing the pellet. Draw the liquid up slowly and carefully, ensuring that no liquid enters the bulb.

8. Dry the samples completely under a light stream of nitrogen gas at 37°C.
  ***Note:*** The drying time depends on the volume but typically does not exceed 60 min. If samples are not completely dry after 60 min, refer to the troubleshooting section (problem 3) for guidance.
  a. Once dried, cap the vials and let them cool to room temperature before continuing.
  b. While vials are cooling, set the heating block temperature to 50°C.
    **CRITICAL:** Start by holding the nitrogen nozzle far from the sample, but inside the GC vial. Adjust the gas flow so that the sample surface barely moves. Gradually bring the nozzle closer to the sample until the surface starts to vibrate more, but only slightly. Ensure that the nozzle never touches the sample, as too much force can cause the sample to be blown out.
    ***Note:*** Ensure complete dryness to prevent moisture from damaging the GC instrument, as moisture can cause corrosion, column damage, instrument failure, and sample contamination.
    ***Note:*** The samples can also be frozen at this point and derivatized on another day. If this is done, the samples will have to be re-dried prior to derivatization to ensure no moisture is present in the vials.

### Part 2: Two-step derivatization

**Timing: 4 h, 20 min**

**Figure 2:**
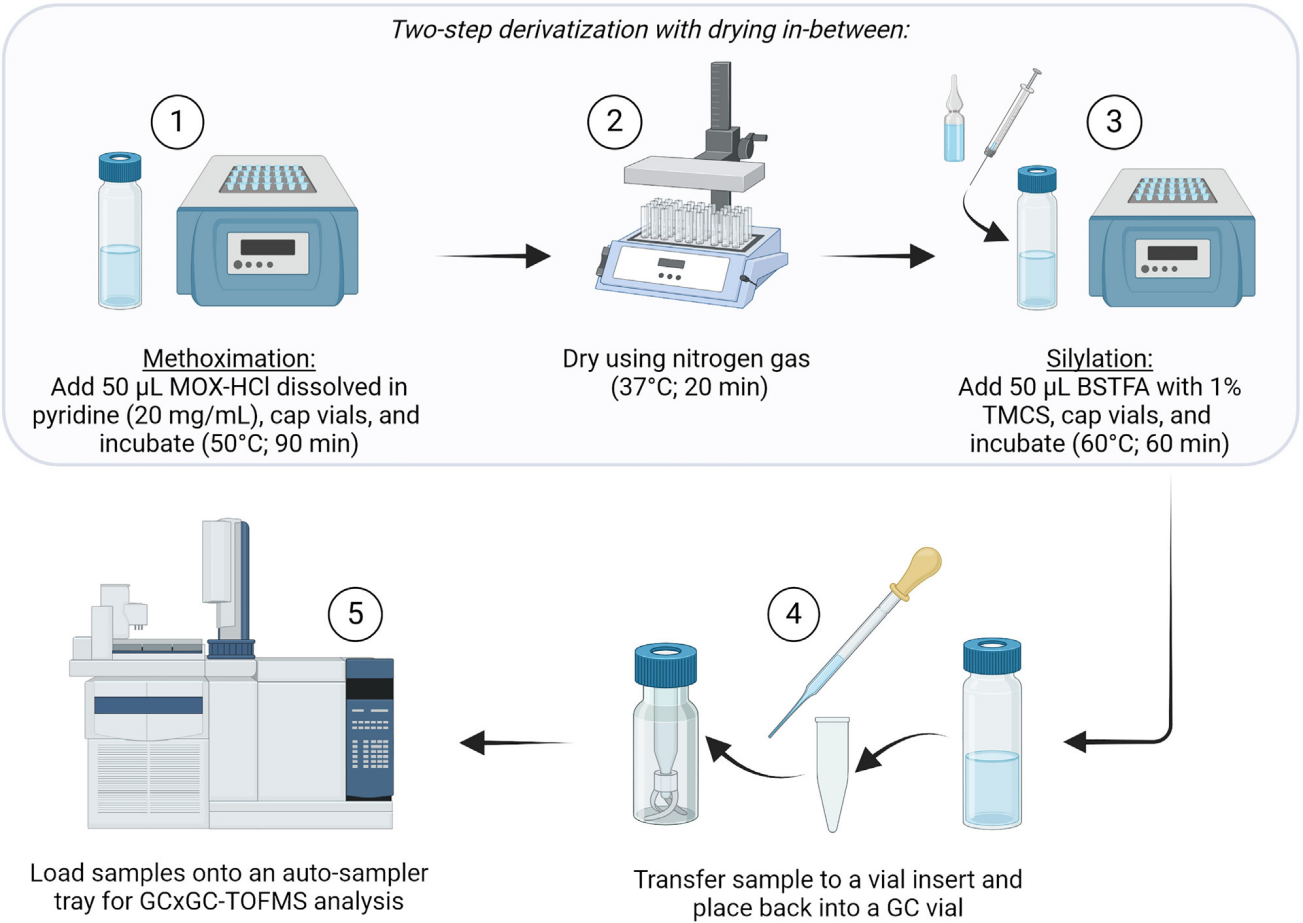
Overview of the two-step derivatization protocol Steps include: (1) Methoximation with methoxyamine hydrochloride (MOX-HCl) at 50°C for 90 min, followed by (2) nitrogen drying, and (3) silylation with N,O‑bis(trimethylsilyl)trifluoroacetamide (BSTFA) with 1% trimethylsilyl chloride (TMCS) at 60°C for 60 min. Hereafter, the samples are (4) transferred to GC vials containing a glass insert, and (5) loaded onto the instrument’s auto-sampler tray. [Created using BioRender.com].

Figure 2 summarizes the two-step derivatization approach, also performed at room temperature (20°C–22°C). When derivatization is performed, samples should be analyzed on a GC within 24 h.

9. Perform methoximation:
  a. Add 50 μL of MOX-HCl dissolved in pyridine (20 mg/mL) to each sample.
  b. Cap the vials, and seal with parafilm.
  c. Incubate these vials by placing them into a Techne Dri-block Heater set at 50°C for 90 min.
  d. Remove the samples and allow to cool to room temperature before continuing.
  e. While vials are cooling, set the heating block temperature to 37°C.

***Note:*** This is a light-sensitive reaction. Switch off the fume hood light but keep the extractor fan on as per safety guidelines.

10. Open the vials and dry the samples again under a light stream of nitrogen gas at 37°C until completely dry. The estimated time for this drying is 20 min.
  a. Before continuing to the next step, remove the samples and set the heating block temperature to 60°C.
11. Perform silylation:
  a. Add 50 μL BSTFA with 1% TMCS to each sample.
  b. Cap the vials and incubate in the Techne Dri-block Heater at 60°C for 60 min.
  c. Remove the samples and allow to cool to room temperature before continuing.
  d. At this point, the heating block can be switched off.

***Note:*** If the sample appears jelly-like as opposed to a clear liquid, refer to the troubleshooting section (problem 4) for guidance.

12. Transfer the samples:
  a. Using a glass Pasteur pipette, transfer the samples to a glass vial insert.
  b. Place this insert into the original (previously used for derivatization) GC vial, and cap.
13. Load the sample vials onto the instrument’s auto-sampler tray.

***Note:*** Prior to loading the samples, it is essential to compile a run-order list detailing the names of the samples and the sequence in which they will be processed. This list should also include the various controls, such as QCs, SBs, and EBs, as well as the FAMEs. Ensure that QCs, EBs and SBs are included in the beginning, middle and end of each sample batch run-order, while FAMEs should be included at the beginning and end of each batch run.

***Note:*** Check the GC vials before analysis to ensure that no condensation has formed, which can be an issue if the auto-sampler tray is cooled. This cooling functionality is instrument-dependent and may not be present on all GC instruments. If water drops appear at the top of the GC cap, use a clean paper towel to wipe the lid and rim, removing any remaining droplets. Once the lid is clean and dry, replace it securely to prevent further contamination or water accumulation.

***Note:*** It may also be necessary to remove the auto-sampler tray from its holder and tip it over a basin to discard any water or condensation that may have formed on the tray. The tray should then also be wiped with a clean paper towel.

***Note:*** Ensure that the sample order in the auto-sampler matches exactly with the pre-determined run-order by double-checking the vial number placements.

### Part 3: GCxGC-TOFMS analysis

**Timing: ≈24 min per sample**

**Figure 3:**
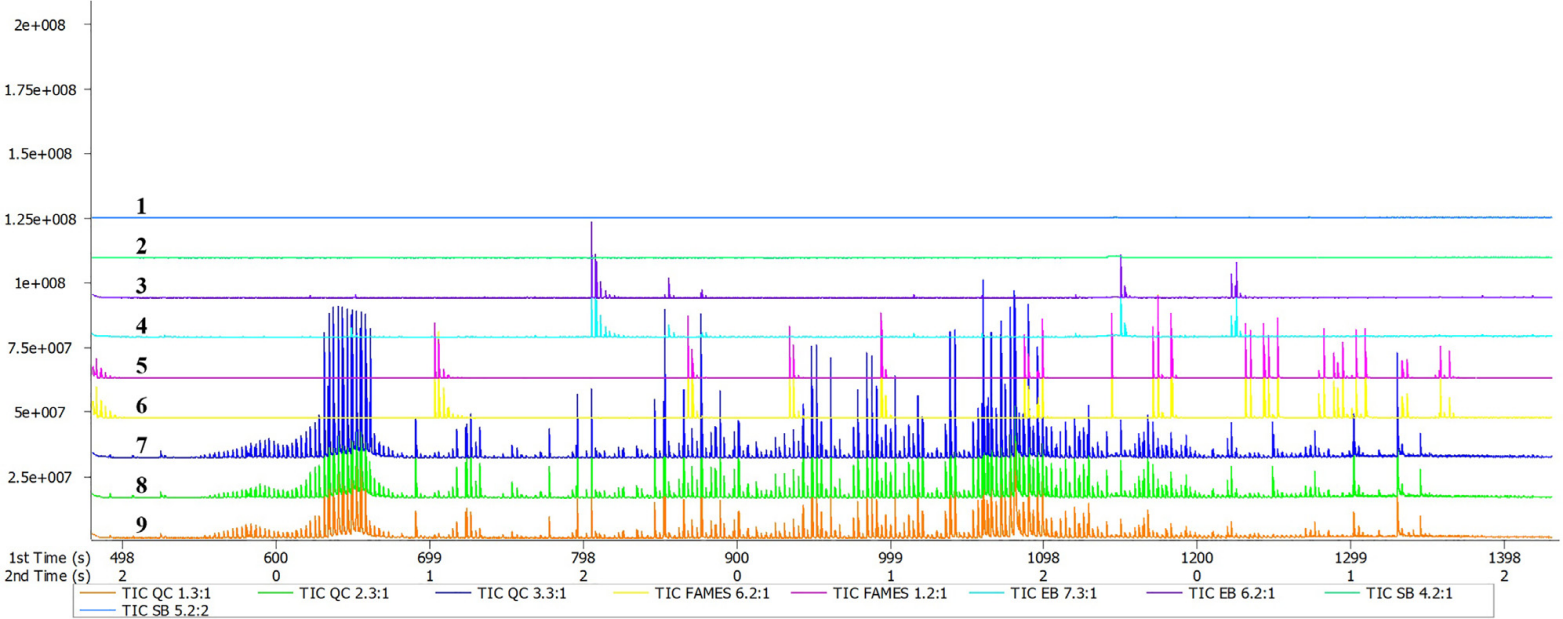
Chromatogram overview Total ion chromatograms (TICs) from the GC-MS analysis of system blanks (no. 1 [blue] and 2 [aqua]), extraction blanks (no. 3 [purple] and 4 [turquoise]), fatty acyl methyl esters (FAMEs; no. 5 [pink] and 6 [yellow]), and quality control (no. 7 [blue], 8 [green], and 9 [orange]) urine samples. This provides an overview of the chemical complexity of the samples.

**Figure 4:**
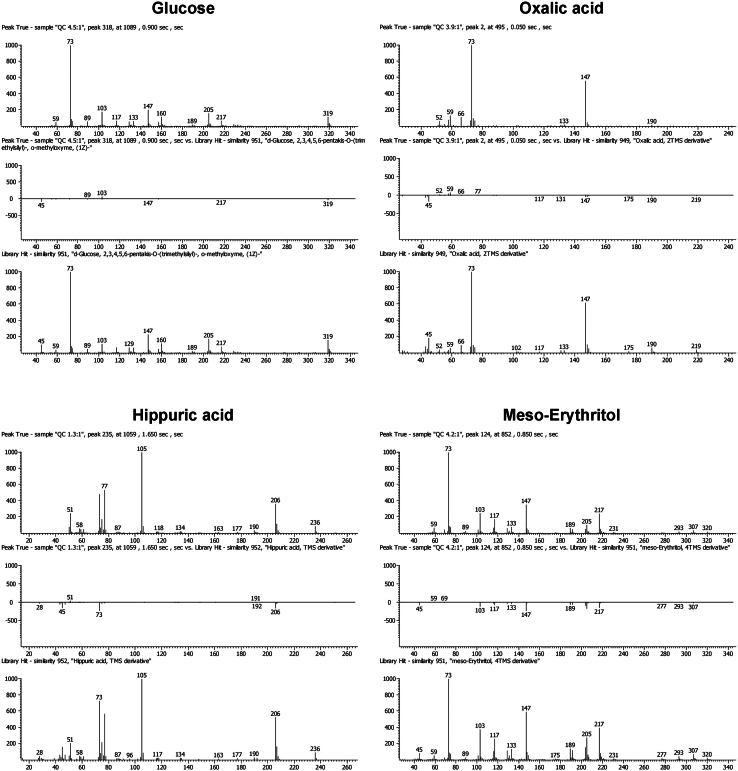
Mass spectral patterns Mass spectral fragmentation patterns of four key compounds identified during the analysis.

This section outlines the setup and parameters for the LECO Pegasus 4D GCxGC-TOFMS system, which is advantageous for analyzing complex mixtures. It includes optimized injection, GC and MS settings, and data processing protocols. Figure 3 shows the total ion chromatogram (TIC) obtained from sample injections using this protocol. Figure 4 presents the fragmentation patterns of key compounds identified, highlighting the mass-to-charge ratios and fragmentation behavior within the mass spectrometer.

14. Use a LECO Pegasus 4D GCxGC-TOFMS fitted with an Agilent 7890 GC system and TOFMS (LECO Africa). The system should have a Restek Rxi-5Sil primary capillary column (28.2 m; 250 μm diameter; 0.25 μm film thickness), and a Restek Rxi-17 secondary capillary column (1.3 m; 250 μm diameter; 0.25 μm film thickness).

***Note:*** While a standard GC-TOFMS system can replace a 2D-GC system for this protocol, we recommend using a 2D-GC for non-targeted studies, if available, due to its superior peak capacity, resolution, and sensitivity, which enhance the separation and detection of complex mixtures, such as urine.

***Note:*** While the system manuals are provided with purchase, further general guidance can be accessed at https://go.leco.com/lecolearn.

15. Inject 1 μL of the sample onto the instrument using a 1:10 split ratio.

***Note:*** The 1:10 split ratio means that only one-tenth of the injected sample enters the column. This helps prevent overloading the column with urine, which is a complex matrix and could overwhelm the system if too much is injected. Sample dilution could be considered as an alternative to split-mode runs. While we have not tested this approach, dilution can also reduce sample complexity and prevent column overloading. Suitable diluents for GC applications include non-polar solvents like hexane or ethyl acetate. It is crucial to choose a diluent that does not interfere with the GC analysis. If adopting this method, it is advisable to validate it to ensure it maintains the sensitivity and resolution of the analysis.

16. Set the GC, MS and DP settings as outlined in the following table:

**Table:** 

Parameter	Setting/Description
**GC Parameters**	

GC Column (Primary)	Restek Rxi-5Sil, 28.2 m, 250 μm ID, 0.25 μm film thickness
GC Column (Secondary)	Restek Rxi-17, 1.3 m, 250 μm ID, 0.25 μm film thickness
Carrier Gas	Purified helium at 1.4 mL/min (constant flow)
Inlet Septum Purge Flow	3 mL/min
Injection Volume	1 μL
Injection Mode	Split (1:10 split ratio)
Mass Selection for Auto Mass Defect Tracking	Exclude masses 149, 207, and 281. ***Note:*** Excluding these masses improves analysis accuracy. Mass 149 is linked to alkyl phthalate contaminants, mass 207 often reflects background noise or column bleed, and mass 281 may indicate silylation byproducts or irrelevant fragments.
Gas Saver Flow Rate	Option on; 15 mL/min, with a front inlet gas save timer set at 1 min
Front Inlet Temperature	250°C **CRITICAL:** This is a critical parameter in GC-MS analysis. It directly impacts analyte volatilization, transfer efficiency, and overall chromatographic performance. ***Note:*** This temperature should be adjusted based on the volatility of the analytes and the injection mode. Optimal settings promote complete volatilization of compounds without causing decomposition, ensuring better analyte desorption and peak resolution. Lower inlet temperatures may be required for thermally labile compounds to prevent degradation, while higher temperatures can be beneficial for less volatile compounds to improve transfer efficiency. ***Note:*** Care must be taken when setting high inlet temperatures, as they can induce thermal degradation of sensitive analytes, affecting both quantitative and qualitative outcomes. Additionally, complex biological matrices may demand more nuanced temperature programs to balance the requirements of diverse compounds, which can be constrained by a fixed inlet temperature*.*
Oven Temperature Program (Primary)	Initial: 70°C for 1 min, then ramp as follows: 5°C/min to 100°C (hold 0 min), 10°C/min to 160°C (hold 0 min), 13°C/min to 230°C (hold 0 min), 20°C/min to 300°C (hold 2 min)
Oven Temperature Program (Secondary)	Enable the secondary oven, and set this to a temperature offset of +5°C. This means that the secondary temperature ramping will be identical to that of the primary oven, but with a 5°C increase at all time points.
Modulator	Enabled for entire run, and set this to 15°C. • Purge pulse time set at 0 s. • Modulation period set to 3 s, with a hot pulse set at 0.7 s, and cool time between stages set at 0.8 s. • Enable the chiller, set at −80°C.
Transfer Line Temperature	225°C **CRITICAL:** This temperature is a critical parameter as it affects the efficient transfer of analytes from the GC to the MS. It is recommended to set the transfer line temperature slightly lower than the inlet temperature to improve overall analytical performance. ***Note:*** Maintain a consistent temperature that aligns with analyte volatility and column compatibility. This helps prevent thermal degradation and excessive column bleed, ensuring optimal performance and accurate results.
Run Time Per Sample	24 min

**MS Parameters**	

MS Ion Source Temperature	200°C • Set the instrument to wait for the ion source temperature to reach the set point before starting acquisition. • Set the source temperature equilibrium time to 0 s.
Mass Range	50–800 m/z
Acquisition Rate	20 spectra per second
Filament Bias (Electron Energy)	−70 eV, with optimized the detector voltage at an offset of 50 V, with a mass defect of 0 mu/100u.
Solvent Delay	480 s ***Note:*** This refers to the length of time from injection until the data system will start storing data from the mass spectrometer; referred to as the “solvent delay”. Although no mass spectra are recorded during this time, this interval is appended to the time axis on the GC column to reflect accurate retention times*.*
MS Run Time	Matches GC run time (24 min)
Masses to Display During Acquisition	“t” (TIC; total ion chromatogram)

**Data Processing (DP) Settings, Using ChromaTOF (LECO) Software**	

MS Library	Mainlib, Replib, NIST, and in-house library
Baseline Tracking	Entire run, with a baseline offset to 1, and the number of data points to average for smoothing set to 3.
Expected Peak Width (Primary Column)	3 s
Expected Peak Width (Secondary Column)	• Match required to combine: 800 (i.e., 80% certainty). • Override the allowed second dimension RT shift to combine early (0.1) and late (0). • Expected peak width: 0.1 s. • Signal-to-noise ratio (S/N): 100. • Integration approach: Traditional.
Maximum Number of Unknown Peaks	100,000
Segmented Processing	From the start to the end of the run, enable peak finding, with an S/N threshold of 300. Include all masses (denoted by ∗), and specify the number of apexing masses as 2.
Common Masses in Derivatized Products	73, 75, 147
Library Parameters	• Library identify search mode: Normal. • Library search mode: Forward at 10. • Masses to library search: ∗ (referring to “all masses collected”). • Minimum molecular weight allowed: 50. • Maximum molecular weight allowed: 800. • Mass threshold: 10. • Similarity match before name is assigned: 800 (this refers to an 80% certainty; we recommend not decreasing this value below 700 or 70%). **CRITICAL:** An 80% similarity match score (800) is used to ensure high confidence in metabolite identification in non-targeted GC-MS metabolomics. This threshold helps standardize results across studies and improves reproducibility by reducing the risk of false positives. Given the complexity of biological samples, a high similarity score supports the biological relevance of identified compounds and facilitates accurate interpretation of metabolic profiles*.*
Libraries tor Searching	Add the NIST (National Institute of Standards and Technology) mass spectral library, which includes two sub-libraries: “mainlib” and “replib”. Additionally, include any relevant in-house libraries, such as those for internal standards used (e.g., “3-Phenylbutyric acid”).
Mass to Use for Area/Height Calculation	“dt” (allow skimming of small riding peaks)
Statistical Compare	• Spectral mass threshold of 10, with a minimum similarity match of 800. • Retention time match criteria, with a maximum number of modulation periods apart of 1 and a maximum retention time difference of 0 s. • Searching for peaks not found by initial peak finding, with a signal-to-noise ratio of 100. • Define analytes to keep, setting the minimum number of samples that contain the analyte to 1, and the minimum percentage of samples in a class that contain the analyte to 1.

## Expected outcomes

**Figure 5:**
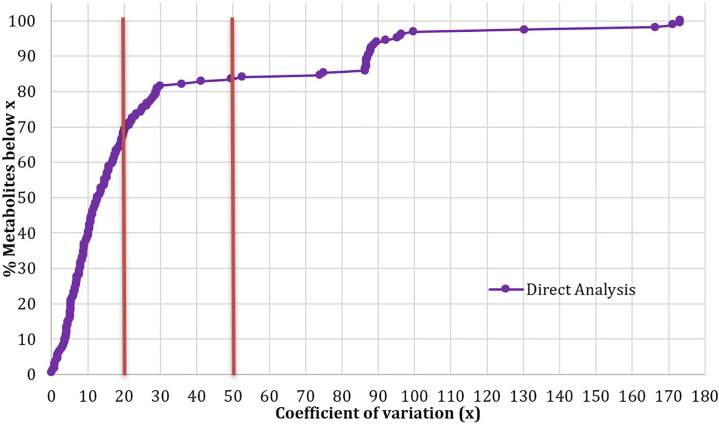
Repeatability of metabolite detection using a 1:10 split on a GCxGC-TOFMS The graph shows the percentage of metabolites detected below the coefficient of variation (CV).

The coefficient of variation (CV) is a statistical measure that expresses the standard deviation as a percentage of the mean. It provides a standardized way to compare the variability of a dataset, regardless of the units or magnitude of the values. A lower CV indicates higher precision, as it suggests that the measurements are clustered more closely around the mean. The Federal Drug Administration (FDA) guidelines for targeted GC-MS analysis recommend a CV of less than 20% as the benchmark for acceptable method repeatability. This means that for targeted analyses, where a specific list of compounds is being measured, the variability of the measurements should not exceed 20% of the mean value. However, in the field of non-targeted metabolomics, where a wide range of compounds are analyzed simultaneously, a higher CV is often accepted. Due to the inherent complexity and diversity of the metabolites being measured, a CV of less than 50% is considered acceptable for non-targeted metabolomics studies.^8^ This higher threshold acknowledges the challenges associated with analyzing a broader spectrum of compounds and the need to balance method precision with the ability to detect a wide range of metabolites. Figure 5 illustrates the repeatability results of our direct analysis protocol for the percentage of metabolites detected below the CV thresholds of 20% and 50% (indicated by red lines) using a 1:10 split on a GCxGC-TOFMS. The results show that 69% of metabolites displayed a CV below 20%, demonstrating a high level of repeatability in the analysis. Furthermore, 83% of the compounds exhibited a CV below 50%, suggesting a substantial degree of reproducibility in non-targeted metabolomics.

**Figure 6:**
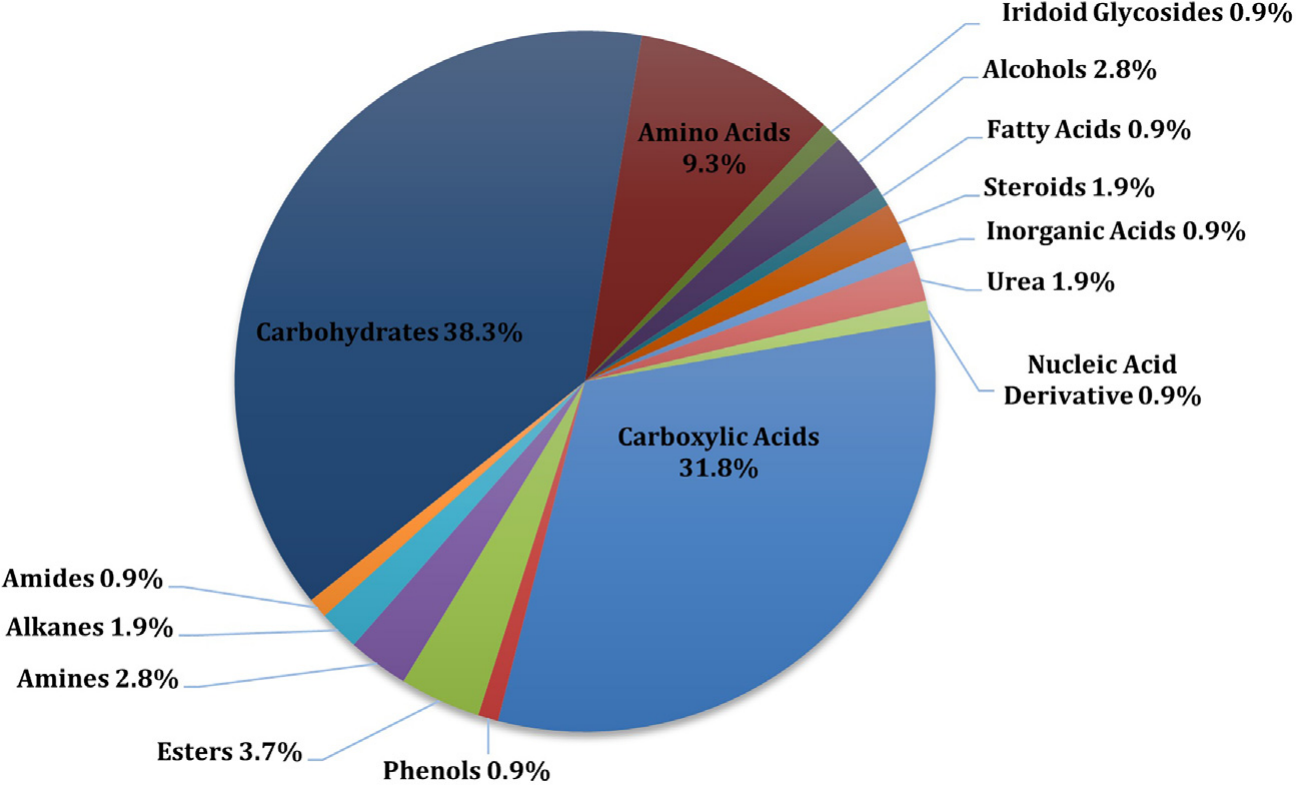
Metabolome coverage of compound classes, excluding unknowns/unannotated compounds, using our direct analysis protocol for non-targeted GCxGC-TOFMS The graph depicts the percentage of each compound class detected, highlighting the highest detection percentages for carbohydrates (38.3%), carboxylic acids (31.8%), and amino acids (9.3%).

Following basic data cleanup, our direct analysis protocol can detect approximately 190 compounds across fifteen different compound classes (see Figure 6), showcasing the diversity of compounds analyzed. Notably, 43.7% of the detected compounds were annotated as “unknown” due to the high similarity matching that did not align with the libraries used. When excluding these unknowns, the top five detected classes were carbohydrates (38.3%), carboxylic acids (31.8%), amino acids (9.3%), esters (3.7%), and alcohols (2.8%). A full list of compounds, along with their unique mass and compound class, is available in the supplementary material (Data S1). For more information on how this protocol compares to other more traditional methods, please refer to Olivier et al.^1^ with the data reported in this paper available via BioStudies: S-BSST1138.

GC-MS is biased toward the analysis of low molecular weight, volatile, thermally stable, and non-polar molecules. This limitation can be addressed by derivatization, which chemically modifies compounds to enhance their suitable for GC analysis by increasing their volatility, decreasing their boiling points, and reducing their polarity.^1^ Derivatization also enhances the detectability of compounds (e.g., steroids or cholesterol) and can increase their stability and sensitivity. This process reduces the complexity of the separation process and enhances the resolution of these compounds.^9^ As a result, the enhanced volatility and thermal stability of the most abundant metabolites excreted in urine, such as carbohydrates and carboxylic acids, leads to a higher metabolome coverage detected by the GC system. In contrast, amino acids are less frequently detected in GC-MS analysis due to their higher polarity and the need for derivatization, which can be less efficient. Additionally, esters and alcohols may be present in lower concentrations in urine samples, leading to their reduced detection. These limitations and the characteristics of GC-MS collectively contribute to the varied detection efficacy of metabolites across different classes.

## Quantification and statistical analysis

After GC-MS analysis, raw data is obtained in .csv format. This file is then converted into an Excel Workbook (.xlsx) for further processing. To convert the .csv file, open it (which will open in Excel by default), and save this as an .xlsx file by selecting “File”, “Save as” and choosing the .xlsx format.

Within the .xlsx file (using Excel), the peak areas in the data must first undergo blank correction procedures before normalization. The data cleanup process involves system blank and extraction blank corrections to identify and exclude signals originating from non-biological sources, as these may cause misinterpretation of results.^7^

Next, data is normalized using the internal standard (IS), which was added at a known volume and concentration to each sample. Normalization is achieved by taking the peak area of each compound within a given sample, dividing it by the peak area of the IS in the same sample, and then multiplying by the concentration factor of the IS added to each sample. This process is repeated for each compound within each individual sample to ensure accurate normalization. For example, in this protocol, 50 ppm IS was added to 100 μL of urine, resulting in a concentration of 50 ng IS/μL urine, assuming a urine density of 1 g/mL. To determine the moles of IS added to the sample, divide the mass (50 ng) by the molar mass of the IS (3-phenylbutyric acid; 164.2 g/mol), giving a concentration factor of 3.04 μmol/L.

After IS normalization, each compound’s concentration must be normalized for the creatinine value of each sample. This is done by taking the concentration (μmol/L) calculated above for each compound within a given sample and dividing it by the creatinine value of the sample, typically provided in mmol/L. The final concentration of each compound is thus expressed as μmol/mmol creatinine (or μmol/μmol creatinine, depending on the preferred units). This two-step normalization process ensures accurate and consistent quantification across all samples. Performing normalization before further data cleanup ensures that metabolite concentrations are adjusted for variations in sample concentration and instrument response, providing a more accurate representation of the data. Further data cleanup, performed after normalization, helps identify and exclude signals from non-biological sources to prevent misinterpretation.

The exact order of the following steps may need to be adjusted depending on the specific dataset. However, for a straightforward two-group comparison, the cleanup steps typically proceed as follows: A 50% zero filter is applied (in Excel) to exclude spurious or infrequently detected compounds, minimizing noise and variability. This filter calculates the percentage of zero values across all samples in a group for each compound, and compounds that are zero in over 50% of a group’s samples are excluded (removed). This step reduces noise and improves statistical power by focusing on consistently detected compounds. Depending on the dataset and the study’s aim, non-derivatized compounds can also be removed from the dataset.

Next, batch effects are assessed using the QC samples. Principal component analysis (PCA) is performed using MetaboAnalyst, which requires converting the Excel Workbook (.xlsx) into a .csv file. PCA visually inspects QC clustering patterns across batches, and if significant batch effects are observed (e.g., QC samples which do not cluster together), corrections are applied using either the batch correction tool in MetaboAnalyst or manual quantile normalization in Excel.

Finally, a 50% quality control-coefficient of variation (QC-CV) filter is applied, removing compounds with CV values exceeding 50% due to significant variability, which could compromise the data.

Once the data cleanup is complete, the dataset is saved as a .csv file for further analysis in MetaboAnalyst, a web-based platform designed for comprehensive metabolomics data analysis, facilitating the exploration and interpretation of metabolomic datasets.

## Limitations

A non-targeted metabolomics approach aims to comprehensively analyze the metabolic profile of a biological system.^10^ This methodology involves the simultaneous identification and quantification of diverse classes of compounds, where the presence of undetected compounds could potentially provide valuable insights. A limitation of this protocol is that it provides metabolite quantities in arbitrary units rather than absolute concentrations. This is a common practice in metabolomics due to the ability to analyze numerous metabolites simultaneously, facilitating the identification of relative differences across samples, which is advantageous for exploratory studies and biomarker discovery. However, absolute quantification is challenging and typically requires additional targeted methods, making this approach less suitable for applications requiring precise metabolite concentrations, such as clinical or pharmacological studies.

While GC systems are widely employed for compound analysis, they have inherent limitations in detecting non-volatile and thermally unstable compounds, such as amino acids. Compounds require derivatization before GC analysis to facilitate their separation and detection. It is crucial to note that derivatization can increase the molecular mass of compounds, potentially exceeding the mass spectrometer’s detection limit and compromising the accuracy of results.^11^

Environmental factors also pose challenges to the protocol’s reliability. For instance, the presence of water in commonly used solvents, like ACN, can affect extraction efficiency and lead to compound loss. Despite advancements in sample preparation techniques, which are generally quick, easy, and affordable, there remains a risk of analyte loss due to protein binding or instability.^12^ Additionally, urease pre-treatment can introduce potential interference, as urease activity may catalyze reactions altering metabolite stability or introducing artifacts. To mitigate this, careful control during sample handling or pre-treatment is essential, especially in studies requiring precise metabolic profiling.^1^ Researchers should carefully evaluate the effects of urease pre-treatment and assess its impact on the reliability and validity of the results to ensure accurate findings. If urease pre-treatment is necessary, sonication is recommended due to its efficiency, uniformity, ease of use, and ability to prevent thermal degradation.^13^^,^^14^

Moreover, repeated freeze-thaw cycles of urine can lead to metabolite degradation. Minimizing these cycles is essential for preserving sample integrity.^15^ The timing of urine collection can also impact metabolite concentrations due to circadian rhythms. First-morning void samples are generally preferred for their higher metabolite concentrations; however, they may not fully reflect the overall metabolic state throughout the day.^16^ Adjusting for urine dilution in certain conditions, like diabetes insipidus, by normalizing metabolite concentrations to creatinine levels might be needed to obtain reliable data. These considerations underscore the importance of consistent sample collection and handling to ensure accurate and reliable results.^17^

Mechanical aspects, like maintaining precise temperature control during GC-MS analysis, are critical for achieving accurate separation and detection of metabolites. Any deviations from optimal temperature profiles can compromise the robustness and reproducibility of the analytical results.^1^ Nevertheless, this direct analysis protocol excels in simultaneously identifying multiple compound classes with high metabolite recovery, making it well-suited for high-throughput sample analysis.

## Troubleshooting

### Problem 1

Internal standard not dissolving (see Preparation 3, point 11a).

### Potential solution

If the internal standard is not dissolving in the Milli-Q water, try the following.

• Sonicate for 20 min at room temperature (20°C–22°C) with high (005) power.
• Add a drop of 5 N sodium hydroxide (NaOH) and swirl the flask again to dissolve.
• If the standard did not dissolve after the addition of NaOH, sonicate again at room temperature with high power for 20 min or until the standard is completely dissolved.

### Problem 2

MOX-HCl not dissolving (see Preparation 3, point 12).

### Potential solution

If the MOX-HCl is not dissolving in the pyridine, try the following.

• Leave for approximately 20 min in the fume hood with the light off and the extractor fan on.
• Place into a GC-MS vial, cap, and seal with parafilm, before sonicating for 20 min at room temperature (20°C–22°C) with high (005) power.

### Problem 3

If drying urine samples using nitrogen gas exceeds the allocated time (see Part 1, point 8).

### Potential solution

If the drying time is insufficient, adjust the distance between the drying nozzle and sample surface, and extend the drying time in increments of 15 min and until completely dry. Monitor closely to avoid over-drying, maintaining a gentle, consistent nitrogen stream.

### Problem 4

Jelly-like consistency of samples after silylation (see Part 2, point 11).

### Potential solution

If the sample appears jelly-like after silylation, add an additional 10 μL of pyridine, mix thoroughly by slightly swirling the vial by hand, and assess the consistency. Repeat the process, if required, until the sample transitions from a jelly-like texture to a fluid, pourable consistency.

## Resource availability

### Lead contact

Further information and requests for resources and reagents should be directed to and will be fulfilled by the lead contact, Laneke Luies (laneke.luies@nwu.ac.za).

### Technical contact

Questions about the technical specifics of performing the protocol should be directed to and will be fulfilled by the technical contact, Laneke Luies (laneke.luies@nwu.ac.za).

### Materials availability

This study did not generate new unique reagents.

### Data and code availability

The accession number for the original/source data reported in this paper is BioStudies: S-BSST1138.

## Acknowledgments

This work is based on the research supported wholly/in part by the National Research Foundation (NRF) of South Africa (grant no. 129871). All opinions, findings and conclusions, or recommendations expressed in this protocol are those of the authors, and the NRF accepts no liability whatsoever in this regard.

## Author contributions

L.L. conceptualized the manuscript; B.A. and L.L. drafted the paper (including all figures). B.A. completed all experimental work under the guidance of L.L. L.L. critically reviewed the content. All authors approved the final version to be submitted. The authors share dual primary co-authorship.

## Declaration of interests

The authors declare no competing interests.

## Supplemental information

Data S1. List of compounds detected using our direct analysis protocol, related to expected outcomes

## References

[1] Olivier C., Allen B., Luies L. (2023). Optimising a urinary extraction method for non-targeted GC–MS metabolomics. Sci. Rep..

[2] Collino S., Martin F.P.J., Rezzi S. (2013). Clinical metabolomics paves the way towards future healthcare strategies. Br. J. Clin. Pharmacol..

[3] Gupta-Wright A., Peters J.A., Flach C., Lawn S.D. (2016). Detection of lipoarabinomannan (LAM) in urine is an independent predictor of mortality risk in patients receiving treatment for HIV-associated tuberculosis in sub-Saharan Africa: a systematic review and meta-analysis. BMC Med..

[4] Christou C., Gika H.G., Raikos N., Theodoridis G. (2014). GC-MS analysis of organic acids in human urine in clinical settings: a study of derivatization and other analytical parameters. Journal of chromatography B.

[5] Olivier C., Luies L. (2024). Metabolic insights into HIV/TB co-infection: An untargeted urinary metabolomics approach. Metabolomics.

[6] Giampietro O., Penno G., Clerico A., Cruschelli L., Cecere M. (1993). How and how long to store urine samples before albumin radioimmunoassay: a practical response. Clin. Chem..

[7] Broadhurst D., Goodacre R., Reinke S.N., Kuligowski J., Wilson I.D., Lewis M.R., Dunn W.B. (2018). Guidelines and considerations for the use of system suitability and quality control samples in mass spectrometry assays applied in untargeted clinical metabolomic studies. Metabolomics.

[8] Schoeman J.C., Du Preez I., Loots D.T. (2012). A comparison of four sputum pre-extraction preparation methods for identifying and characterising M. tuberculosis using GCxGC-TOFMS metabolomics. J. Microbiol. Methods.

[9] Ghayth G.M. (2021). Application of GC in the Analysis of Carbohydrates. Academic Journal of Research and Scientific Publishing|.

[10] Shulaev V. (2006). Metabolomics technology and bioinformatics. Brief. Bioinform..

[11] Villiers L., Loots D. (2014). Using metabolomics for elucidating the mechanisms related to tuberculosis treatment failure. Curr. Metabolomics.

[12] Polson C., Sarkar P., Incledon B., Raguvaran V., Grant R. (2003). Optimization of protein precipitation based upon effectiveness of protein removal and ionization effect in liquid chromatography-tandem mass spectrometry. J. Chromatogr. B Analyt. Technol. Biomed. Life Sci..

[13] Taufik M., Ardilla D., Cahyady B., Alfian Z., Razali M., Susilawati E., Afniwati A. (2023). Sonication technique for nicotine extraction from saliva and urine. AIP Conf. Proc..

[14] Kim J., Choi K., Chung D.S. (2012). Sample Preparation for Capillary Electrophoretic Applications. Comprehensive Sampling and Sample Preparation.

[15] Rotter M., Brandmaier S., Prehn C., Adam J., Rabstein S., Gawrych K., Brüning T., Illig T., Lickert H., Adamski J., Wang-Sattler R. (2017). Stability of targeted metabolite profiles of urine samples under different storage conditions. Metabolomics.

[16] González-Domínguez R., González-Domínguez Á., Sayago A., Fernández-Recamales Á. (2020). Recommendations and Best Practices for Standardizing the Pre-Analytical Processing of Blood and Urine Samples in Metabolomics. Metabolites.

[17] Ahloulay M., Schmitt F., Déchaux M., Bankir L. (1999). Vasopressin and urinary concentrating activity in diabetes mellitus. Diabetes Metab..

