# Supplementary material for: Protocol for a low-volume, direct analysis urine preparation procedure for non-targeted GC-MS metabolomics
Source: STAR Protoc. 2024 Nov 15;5(4):103449. doi: 10.1016/j.xpro.2024.103449 (PMC11609456; doi:10.1016/j.xpro.2024.103449)
Supplement: Data S1. List of compounds detected using our direct analysis protocol, related to expected outcomes [file mmc1.pdf]

LIST OF COMPOUNDS DETECTED USING OUR DIRECT ANALYSIS PROTOCOL

COMPOUND (UNIQUE MASS)	CLASS
3-Phenylbutyric acid-TMS (IS) (UM:118)	**Internal standard**
Glycerol, tris(trimethylsilyl) ether [1] (UM:205)	Alcohols
Glycerol, tris(trimethylsilyl) ether [2] (UM:205)	Alcohols
meso-Erythritol, tetrakis(trimethylsilyl) ether (UM:217)	Alcohols
3,8-Dioxa-2,9-disiladec-5-ene, 2,2,9,9-tetramethyl-, (E)- (UM:247)	Alkanes
Butane, 2,3-bis(trimethylsiloxy)- (UM:117)	Alkanes
Acetamide, 2,2,2-trifluoro-N-(trimethylsilyl)- (UM:77)	Amides
2-Ethylbutan-N-trimethylsilyl-1-amine (UM:102)	Amines
2-Pentamethyldisilyloxypropane (UM:169)	Amines
Creatinine enol N1,N3,O-tris(trimethylsilyl) (UM:115)	Amines
Alanine 2TMS (UM:116)	Amino Acids
GABA 3TMS (UM:174)	Amino Acids
Glycine, N-(1-oxobutyl)-, trimethylsilyl ester (UM:158)	Amino Acids
Glycine, N-benzoyl-, trimethylsilyl ester (UM:206)	Amino Acids
Leucine 2TMS (UM:158)	Amino Acids
L-Proline, 5-oxo-1-(trimethylsilyl)-, trimethylsilyl ester (UM:156)	Amino Acids
N,O,O-Tris(trimethylsilyl)-L-threonine (UM:57)	Amino Acids
N-Acetyltyrosine, di-TMS (UM:179)	Amino Acids
Phenylalanine (UM:N/A)	Amino Acids
Serine, N,O-bis(trimethylsilyl)-, trimethylsilyl ester (UM:204)	Amino Acids
á-D-Galactopyranoside, methyl 2,3-bis-O-(trimethylsilyl)-, cyclic methylboronate (UM:69)	Carbohydrates
à-D-Glucopyranoside, methyl 2-(acetylamino)-2-deoxy-3-O-(trimethylsilyl)-, cyclic methylboronate (UM:187)	Carbohydrates
à-D-Glucopyranoside, methyl 2-(acetylamino)-2-deoxy-3-O-(trimethylsilyl)-, cyclic methylboronate (UM:187)	Carbohydrates
D-(-)- Erythrofuranose, tris(trimethylsilyl) ether (isomer 1) (UM:103)	Carbohydrates
D-(-)- Erythrofuranose, tris(trimethylsilyl) ether (isomer 2) (UM:73)	Carbohydrates
D-(-)- Erythrofuranose, tris(trimethylsilyl) ether (isomer 2) (UM:73)	Carbohydrates
D-(-)-Erythrose, tris(trimethylsilyl) ether, methyloxime (anti) (UM:147)	Carbohydrates
D-(-)-Lyxofuranose, tetrakis(trimethylsilyl) ether (UM:217)	Carbohydrates
D-(-)-Lyxofuranose, tetrakis(trimethylsilyl) ether (UM:217)	Carbohydrates
D-(-)-Rhamnose, tetrakis(trimethylsilyl) ether, methyloxime (syn) (UM:160)	Carbohydrates
D-(-)-Tagatofuranose, pentakis(trimethylsilyl) ether (isomer 2) (UM:217)	Carbohydrates
D-(-)-Tagatose, pentakis(trimethylsilyl) ether, methyloxime (syn) (UM:217)	Carbohydrates
D-(+)-Glucuronic acid ç-lactone, tris(trimethylsilyl) ether, methyloxime (anti) (UM:160)	Carbohydrates
D-(+)-Xylose, tetrakis(trimethylsilyl) ether, methyloxime (anti) (UM:103)	Carbohydrates
D-Arabinopyranose, tetrakis(trimethylsilyl) ether (isomer 2) (UM:191)	Carbohydrates
D-Erythronic acid ç-lactone, bis(trimethylsilyl) ether (UM:73)	Carbohydrates
d-Galactose, 2,3,4,5,6-pentakis-O-(trimethylsilyl)-, o-methyloxyme, (1Z)- (UM:319)	Carbohydrates
d-Galactose, 2,3,4,5,6-pentakis-O-(trimethylsilyl)-, o-methyloxyme, (1Z)- (UM:319)	Carbohydrates
d-Galactose, 2,3,4,5,6-pentakis-O-(trimethylsilyl)-, o-methyloxyme, (1Z)- (UM:319)	Carbohydrates
D-Gluconic acid, 2,3,4,5,6-pentakis-O-(trimethylsilyl)-, trimethylsilyl ester (UM:147)	Carbohydrates
d-Glucose, 2,3,4,5,6-pentakis-O-(trimethylsilyl)-, o-methyloxyme, (1Z)- (UM:160)	Carbohydrates
D-Glucuronic acid, 2,3,4,5-tetrakis-O-(trimethylsilyl)-, trimethylsilyl ester (UM:217)	Carbohydrates
Glycoside, à-methyl-trtrakis-O-(trimethylsilyl)- (UM:204)	Carbohydrates
Inositol, 1,2,3,4,5,6-hexakis-O-(trimethylsilyl)-, scyllo- (UM:318)	Carbohydrates
L-(+)-Threose, tris(trimethylsilyl) ether, methyloxime (syn) (UM:147)	Carbohydrates
Lyxose, tetra-(trimethylsilyl)-ether (UM:217)	Carbohydrates
Myo-Inositol, 1,2,3,4,5,6-hexakis-O-(trimethylsilyl)- [1] (UM:318)	Carbohydrates
Myo-Inositol, 1,2,3,4,5,6-hexakis-O-(trimethylsilyl)- [2] (UM:147)	Carbohydrates
Myo-Inositol, 1,2,3,4,5,6-hexakis-O-(trimethylsilyl)- [miss ID] (UM:73)	Carbohydrates
Pentitol, 1-desoxytetrakis-O-(trimethylsilyl)- (UM:117)	Carbohydrates

Ribitol (oop) 5TMS (UM:103)	Carbohydrates
Ribitol, 1,2,3,4,5-pentakis-O-(trimethylsilyl)- (UM:217)	Carbohydrates
Ribitol, 1,2,3,4,5-pentakis-O-(trimethylsilyl)- (UM:217)	Carbohydrates
Ribitol, 1,2,3,4,5-pentakis-O-(trimethylsilyl)- (UM:217)	Carbohydrates
Ribitol, 1,2,3,4,5-pentakis-O-(trimethylsilyl)- (UM:217)	Carbohydrates
Ribitol, 1,2,3,4,5-pentakis-O-(trimethylsilyl)- (UM:217)	Carbohydrates
Ribitol, 1,2,3,4,5-pentakis-O-(trimethylsilyl)- (UM:217)	Carbohydrates
Ribitol, 1,2,3,4,5-pentakis-O-(trimethylsilyl)- (UM:217)	Carbohydrates
Ribitol, 1,2,3,4,5-pentakis-O-(trimethylsilyl)- (UM:217)	Carbohydrates
Ribonic acid, 2,3,4,5-tetrakis-O-(trimethylsilyl)-, trimethylsilyl ester (UM:147)	Carbohydrates
Xylo-hexos-5-ulose, 2,3,4,6-tetrakis-O-(trimethylsilyl)-, bis(O-methyloxime) (UM:133)	Carbohydrates
(R*,R*)-2,3-Dihydroxybutanoic acid, tris(trimethylsilyl)- (UM:117)	Carboxylic Acids
1-Propene-1,2,3-tricarboxylic acid, tris(trimethylsilyl) ester, (E)- (UM:229)	Carboxylic Acids
2,3,4-Trihydroxybutyric acid tetrakis(trimethylsilyl) deriv., (, (R*,R*)-) (UM:N/A)	Carboxylic Acids
2,3,4-Trihydroxybutyric-Acid-Lactone (UM:147)	Carboxylic Acids
2,3-Dihydroxybutanoic-Acid (UM:117)	Carboxylic Acids
2-Butenedioic acid (Z)-, bis(trimethylsilyl) ester (UM:245)	Carboxylic Acids
2-Butenedioic acid, 2,3-bis[(trimethylsilyl)oxy]-, bis(trimethylsilyl) ester, (Z)- (UM:333)	Carboxylic Acids
2-Pentenedioic acid, 3-methyl-, bis(trimethylsilyl) ester (UM:82)	Carboxylic Acids
2-Pentenoic acid, 2-[(trimethylsilyl)oxy]-, trimethylsilyl ester (UM:147)	Carboxylic Acids
4-Cyclohexene-1,2-dicarboxylic acid, 4-chloro-, bis(trimethylsilyl) ester (UM:73)	Carboxylic Acids
á-Hydroxypyruvic acid, trimethylsilyl ether, trimethylsilyl ester (UM:133)	Carboxylic Acids
Benzeneacetic acid, 3-methoxy-à,4-bis[(trimethylsilyl)oxy]-, trimethylsilyl ester (UM:297)	Carboxylic Acids
Benzoic acid, 2-[(trimethylsilyl)amino]-, trimethylsilyl ester (UM:266)	Carboxylic Acids
Benzoic acid, 4-[(trimethylsilyl)oxy]-, trimethylsilyl ester (UM:223)	Carboxylic Acids
Butanedioic acid, bis(trimethylsilyl) ester [miss-ID] (UM:73)	Carboxylic Acids
Butanoic acid, 2,4-bis[(trimethylsilyl)oxy]-, trimethylsilyl ester (UM:103)	Carboxylic Acids
Butanoic acid, 3,4-bis[(trimethylsilyl)oxy]-, trimethylsilyl ester (UM:73)	Carboxylic Acids
Citric acid 4TMS (UM:273)	Carboxylic Acids
Citric acid 4TMS [miss ID] (UM:82)	Carboxylic Acids
D-threo-Pentonic acid, 3-deoxy-2,5-bis-O-(trimethylsilyl)-2-C-[[(trimethylsilyl)oxy]methyl]-, lactone [1] (UM:245)	Carboxylic Acids
D-threo-Pentonic acid, 3-deoxy-2,5-bis-O-(trimethylsilyl)-2-C-[[(trimethylsilyl)oxy]methyl]-, lactone [2] (UM:103)	Carboxylic Acids
Erythro-Pentonic acid, 2-deoxy-3,4,5-tris-O-(trimethylsilyl)-, trimethylsilyl ester [1] (UM:69)	Carboxylic Acids
Erythro-Pentonic acid, 2-deoxy-3,4,5-tris-O-(trimethylsilyl)-, trimethylsilyl ester [2] (UM:204)	Carboxylic Acids
Erythro-Pentonic acid, 2-deoxy-3,4,5-tris-O-(trimethylsilyl)-, trimethylsilyl ester [3] (UM:73)	Carboxylic Acids
Ethanedioic acid, bis(trimethylsilyl) ester [miss ID] (UM:73)	Carboxylic Acids
L-Ascorbic acid (UM:N/A)	Carboxylic Acids
Malonic acid, bis(2-trimethylsilylethyl ester (UM:147)	Carboxylic Acids
METHYLMALONIC-ACID-Tritms (UM:245)	Carboxylic Acids
OXALIC-ACID [1] (UM:147)	Carboxylic Acids
Pentanedioic acid, 2-[(trimethylsilyl)oxy]-, bis(trimethylsilyl) ester (UM:129)	Carboxylic Acids
Pentanedioic acid, 3-methyl-, bis(trimethylsilyl) ester (UM:69)	Carboxylic Acids
Propanoic acid, 2-methyl-3-oxo-3-[[2-oxo-2-[(trimethylsilyl)oxy]ethyl](trimethylsilyl)amino]- (UM:158)	Carboxylic Acids
Succinic acid (UM:247)	Carboxylic Acids
trans-3-Hexenedioic acid, bis(trimethylsilyl) ester (UM:274)	Carboxylic Acids
1H-Indole, 1-(trimethylsilyl)-5-[(trimethylsilyl)oxy]- (UM:277)	Esters
2-Ethyl-3-trimethylsilyloxy(trimethylsilyl)butyrate (UM:117)	Esters
p-Trimethylsilyloxyphenyl-(trimethylsilyloxy)trimethylsilylacrylate (UM:267)	Esters
Trimethylsilyl [3-methoxy-4-(trimethylsilyloxy)phenyl]acetate (UM:179)	Esters
Palmitic acid TMS (UM:117)	Fatty Acids
Trimethylsilyl fluoride (UM:77)	Inorganic Acids
Aucubin, hexakis(trimethylsilyl) ether (UM:103)	Iridoid Glycosides

Uric acid, N,O,O',O''-tetrakis(trimethylsilyl)- (UM:441)	Nucleic Acid Derivative
1,2-Benzenediol bis(trimethylsilyl) ether (UM:254)	Phenols
Pregn-4-en-18-al, 3-(methoxyimino)-20-oxo-11,21-bis[(trimethylsilyl)oxy]-, 18-(O-methyloxime), (11á,17à)- (UM:77)	Steroids
Pregn-5-en-20-one, 3,16,17,21-tetrakis[(trimethylsilyl)oxy]-, O-(phenylmethyl)oxime, (3á,16à)- (UM:205)	Steroids
Analyte 100 (UM:125)	Unknown
Analyte 102 (UM:123)	Unknown
Analyte 106 (UM:239)	Unknown
Analyte 115 (UM:129)	Unknown
Analyte 118 (UM:55)	Unknown
Analyte 123 (UM:95)	Unknown
Analyte 125 (UM:171)	Unknown
Analyte 126 (UM:164)	Unknown
Analyte 128 (UM:103)	Unknown
Analyte 13 (UM:73)	Unknown
Analyte 131 (UM:73)	Unknown
Analyte 137 (UM:179)	Unknown
Analyte 14 (UM:102)	Unknown
Analyte 143 (UM:175)	Unknown
Analyte 148 (UM:119)	Unknown
Analyte 150 (UM:175)	Unknown
Analyte 156 (UM:117)	Unknown
Analyte 157 (UM:55)	Unknown
Analyte 167 (UM:175)	Unknown
Analyte 168 (UM:103)	Unknown
Analyte 170 (UM:187)	Unknown
Analyte 176 (UM:147)	Unknown
Analyte 18 (UM:245)	Unknown
Analyte 181 (UM:217)	Unknown
Analyte 192 (UM:117)	Unknown
Analyte 194 (UM:129)	Unknown
Analyte 199 (UM:146)	Unknown
Analyte 20 (UM:89)	Unknown
Analyte 206 (UM:193)	Unknown
Analyte 210 (UM:179)	Unknown
Analyte 212 (UM:119)	Unknown
Analyte 216 (UM:297)	Unknown
Analyte 219 (UM:217)	Unknown
Analyte 225 (UM:298)	Unknown
Analyte 226 (UM:149)	Unknown
Analyte 23 (UM:147)	Unknown
Analyte 239 (UM:175)	Unknown
Analyte 25 (UM:115)	Unknown
Analyte 251 (UM:221)	Unknown
Analyte 255 (UM:147)	Unknown
Analyte 27 (UM:73)	Unknown
Analyte 272 (UM:118)	Unknown
Analyte 275 (UM:383)	Unknown
Analyte 278 (UM:193)	Unknown
Analyte 284 (UM:193)	Unknown
Analyte 288 (UM:133)	Unknown
Analyte 29 (UM:131)	Unknown
Analyte 3 (UM:221)	Unknown
Analyte 302 (UM:136)	Unknown

Analyte 304 (UM:180)	Unknown
Analyte 305 (UM:217)	Unknown
Analyte 306 (UM:159)	Unknown
Analyte 311 (UM:273)	Unknown
Analyte 314 (UM:254)	Unknown
Analyte 317 (UM:73)	Unknown
Analyte 321 (UM:55)	Unknown
Analyte 322 (UM:73)	Unknown
Analyte 323 (UM:73)	Unknown
Analyte 325 (UM:223)	Unknown
Analyte 38 (UM:226)	Unknown
Analyte 4 (UM:207)	Unknown
Analyte 43 (UM:55)	Unknown
Analyte 49 (UM:73)	Unknown
Analyte 5 (UM:147)	Unknown
Analyte 55 (UM:174)	Unknown
Analyte 56 (UM:116)	Unknown
Analyte 58 (UM:174)	Unknown
Analyte 59 (UM:241)	Unknown
Analyte 60 (UM:210)	Unknown
Analyte 70 (UM:73)	Unknown
Analyte 71 (UM:240)	Unknown
Analyte 72 (UM:100)	Unknown
Analyte 74 (UM:147)	Unknown
Analyte 75 (UM:160)	Unknown
Analyte 82 (UM:82)	Unknown
Analyte 85 (UM:131)	Unknown
Analyte 87 (UM:174)	Unknown
Analyte 9 (UM:147)	Unknown
Analyte 90 (UM:70)	Unknown
Analyte 93 (UM:111)	Unknown
Analyte 96 (UM:81)	Unknown
Analyte 98 (UM:143)	Unknown
Analyte 99 (UM:117)	Unknown
Urea, N,N'-bis(trimethylsilyl)- [1] (UM:N/A)	Urea
Urea, N,N'-bis(trimethylsilyl)- [miss ID] (UM:101)	Urea
